# A Smartphone App With a Digital Care Pathway for Patients Undergoing Spine Surgery: Development and Feasibility Study

**DOI:** 10.2196/21138

**Published:** 2020-10-16

**Authors:** Madison Ponder, Abena A. Ansah-Yeboah, Lefko T. Charalambous, Syed M. Adil, Vishal Venkatraman, Muhammad Abd-El-Barr, Michael Haglund, Peter Grossi, Chester Yarbrough, Rajeev Dharmapurikar, Ziad Gellad, Shivanand P. Lad

**Affiliations:** 1 Higgs Boson Health Durham, NC United States; 2 Department of Neurosurgery Duke University Medical Center Durham, NC United States

**Keywords:** development, digital interventions, digital health, smartphone app, surgery, behavior change, mobile app, technology, mobile phone, mHealth

## Abstract

**Background:**

There is a great unmet clinical need to provide patients undergoing spinal surgery and their caregivers with ongoing, high-quality care before and after surgery in an efficiency-focused health care environment.

**Objective:**

The objective of this study is to design, develop, and evaluate the acceptability and feasibility of a novel planning-, outcomes-, and analytics-based smartphone app called ManageMySurgery (MMS) in patients undergoing elective spine surgery (MMS-Spine).

**Methods:**

The development process of the MMS app was conducted over 2 sequential stages: (1) an evidence-based intervention design with refinement from surgeon and patient feedback and (2) feasibility testing in a clinical pilot study. We developed a novel, mobile-based, Health Insurance Portability and Accountability Act–compliant platform for interventional and surgical procedures. It is a patient-centric mobile health app that streamlines patients’ interactions with their care team. MMS divides the patient journey into phases, making it feasible to provide customized care pathways that meet patients’ unique needs. Patient-reported outcomes are easily collected and conform to the National Institutes of Health Patient-Reported Outcomes Measurement Information System (PROMIS) standard.

**Results:**

We tested the feasibility of the MMS-Spine app with patients undergoing elective spine surgery at a large academic health system. A total of 47 patients undergoing elective spine surgery (26 cervical spine and 21 lumbar spine surgeries) downloaded and used MMS-Spine to navigate their surgical journey, quantify their baseline characteristics and postoperative outcomes, and provide feedback on the utility of the app in preparing for and recovering from their spinal surgery. The median age was 59.0 (range 33-77) years, 22 of the 47 patients (47%) were women, and 26 patients (55%) had commercial insurance. Of the 47 patients, a total of 33 (70%) logged in on an iOS device, 11 (23%) on an Android device, and 3 (6%) on a computer or tablet. A total of 17 of the 47 patients (36%) added a caregiver, of which 7 (41%) logged in. The median number of sign-ins was 2. A total of 38 of 47 patients (81%) completed their baseline preoperative PROMIS-29 outcomes, and 14 patients (30%) completed at least one PROMIS-29 survey during the postoperative period. Of the 24 patients who completed the MMS survey, 21 (88%) said it was helpful during preparation for their procedure, 16 (67%) said it was helpful during the postoperative period, and 23 (96%) said that they would recommend MMS to a friend or family member.

**Conclusions:**

We used a patient-centered approach based on proven behavior change techniques to develop a comprehensive smartphone app for patients undergoing elective spine surgery. The optimized version of the app is ready for formal testing in a larger randomized clinical study to establish its cost-effectiveness and effect on patients’ self-management skills and long-term outcomes.

## Introduction

Disorders of the spine are among the most prevalent medical conditions worldwide. In the United States, over US $85 billion is spent annually on spine-related problems, which are the second leading cause of hospital-related visits after the common cold [1]. When conservative options have been exhausted, many patients undergo spine surgery to relieve their pain. Recently, increasing efficiency and cost pressures have significantly impacted postoperative care. Patients are being discharged earlier, and symptoms that would have previously prompted a longer postoperative stay are now being managed remotely. Moreover, without easy access to reliable remote medical information and risk assessment, patients may delay seeking care, experience unnecessary anxiety, or seek unnecessary care. Given the ubiquity of smartphone use, mobile apps are actively being implemented as platforms to connect care providers with patients and provide information and communication outside of a traditional medical office visit.

A number of studies have demonstrated that digital health solutions and patient-reported outcomes (PROs) improve the results of chronic medical conditions [2]. Some mobile apps have been developed to use as perioperative care tools to communicate presurgical and postsurgical instructions and concerns. Feasibility studies for apps for abdominal and orthopedic surgeries have shown that they are convenient for patients to use and can reduce the need for follow-up visits [3-5]. However, there are currently no validated solutions aimed at acute spinal surgical time points, which are among the most stressful health care experiences in the lives of patients and their caregivers. There is a great unmet clinical need to provide patients undergoing surgery and their caregivers with ongoing high-quality care before and after surgery in an increasingly efficiency-focused health care environment. To address this need, we created a novel planning-, outcomes-, and analytics-based platform called ManageMySurgery (MMS), which includes a specific module for spine surgery (MMS-Spine), and conducted feasibility testing in patients undergoing elective spine surgery.

## Methods

### MMS Development

**Figure 1:**
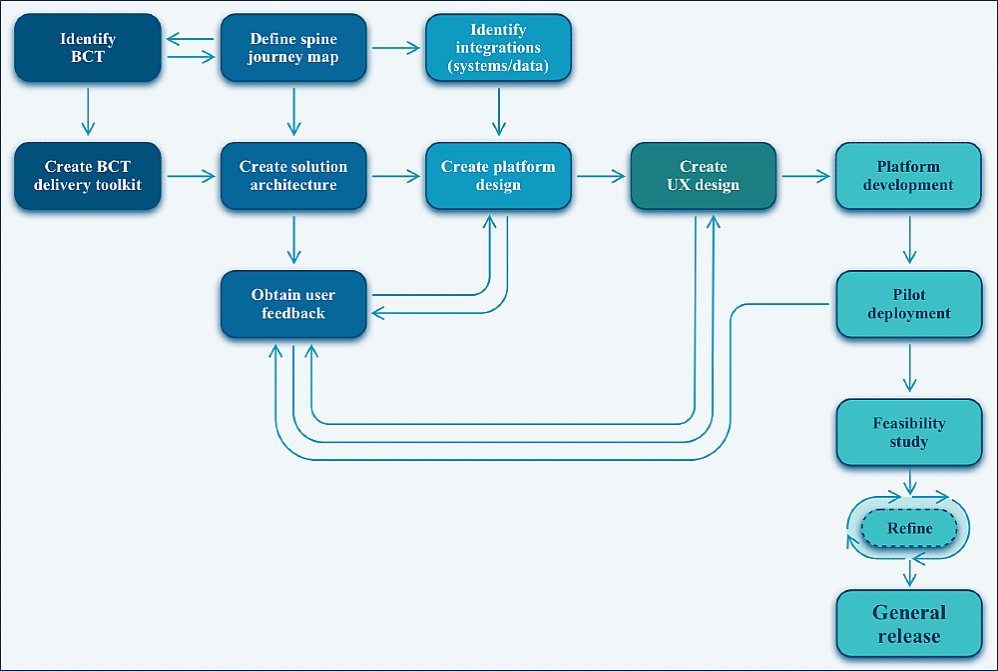
Development overview process of the creation and implementation of the MMS-Spine app. BCT: behavior change technique; MMS-Spine: ManageMySurgery spine surgery module; UX: user experience.

**Figure 2:**
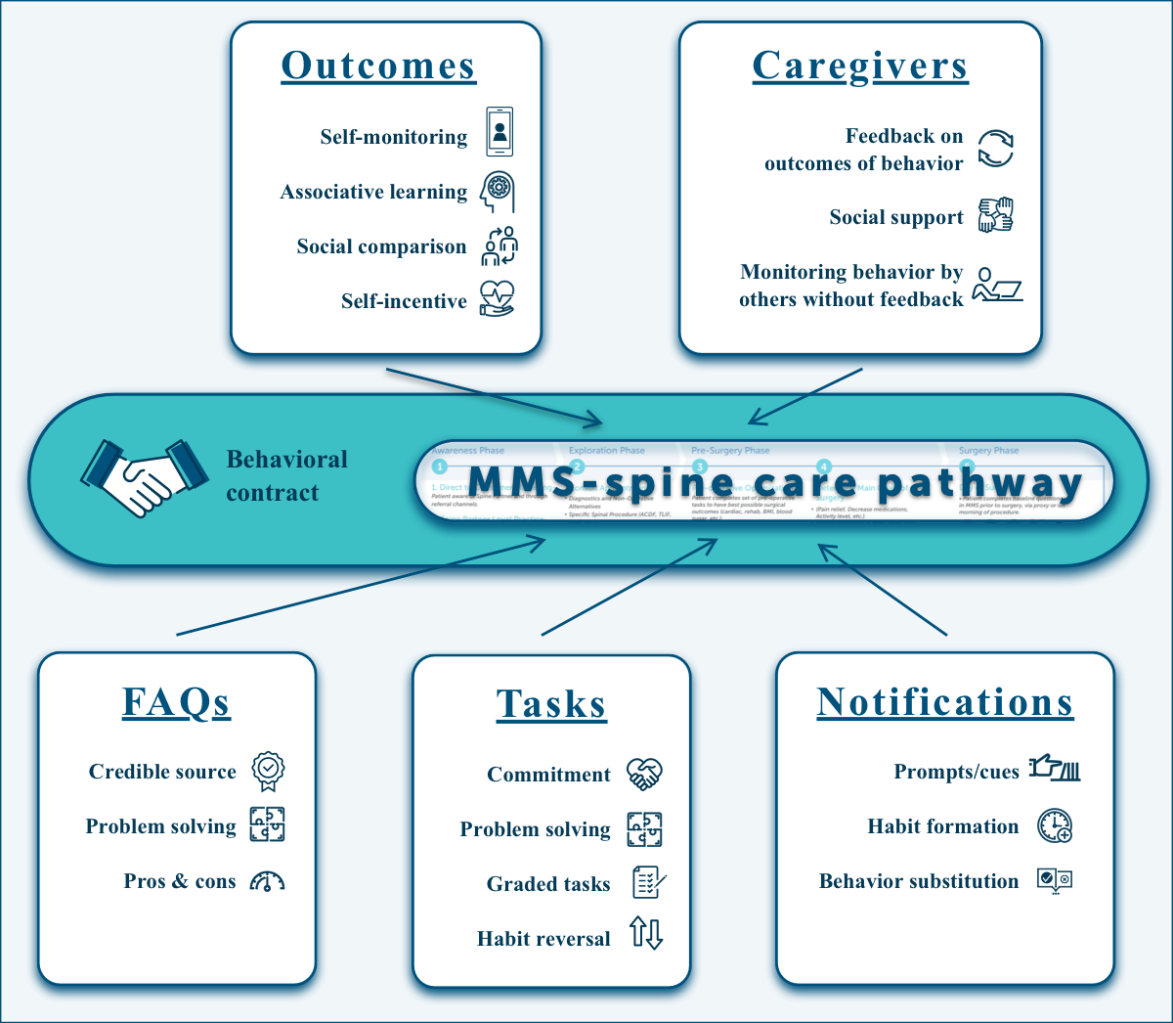
MMS-Spine features incorporating behavior change technique methods applicable to clinical care. FAQ: frequently asked question; MMS-Spine: ManageMySurgery spine surgery module.

We developed the MMS app in 2 stages: (1) an evidence-based intervention design with refinement by health care providers using the MMS app (Figure 1) and (2) feasibility testing in a clinical pilot study. This section presents the procedures and key findings used to inform the next stage of the iterative development process.

### Stage 1: Consultation With Experts, Intervention Design, and Outcomes

#### App Overview

MMS is a cloud-based, Health Insurance Portability and Accountability Act–compliant solution that provides a platform that acts as an extension of the clinical care team for patients undergoing surgical procedures and their caregivers. The goal of MMS is to provide a solution that provides patients and their families the best possible surgical experience while tracking quantifiable outcomes from surgery. MMS does this by providing a way for patients and their caregivers to prepare for procedures and make shared decisions, leading to lower overall anxiety, increased satisfaction, and increased retention of patients in their digital care pathway. In short, better patient engagement and better workflows can lead to overall better outcomes at a lower cost. The app was designed to function on mobile operating systems, including Android (Google Inc) and iOS (Apple Inc), and as a web application to allow for the widest possible use.

Behavior change techniques (BCTs) are designed to enable behavior change by augmenting factors that facilitate behavior change or by mitigating factors that inhibit behavior change [6]. MMS was designed to administer BCTs that fit within the clinical workflow (journey map, frequently asked questions, tasks, notifications, and outcomes; see Figure 2) [6,7].

The design of MMS-Spine was informed by an interdisciplinary group of experts in surgery, behavior change, psychometrics, and computer science. Sources for app content included evidence-based guidelines from national societies that specialize in spine care and surgery (North American Spine Society, American Association of Neurological Surgeons, and the American Association of Orthopedic Surgeons). Importantly, the wording of questions, responses, and other content in the app was developed through an iterative design process with a scientific writing team so that all information was presented at a sixth-grade reading level at maximum. Literacy evaluation was performed by the Duke Patient Education Governance Council. The goals of this process were to make the app accessible and patient centered while also improving communication and patient knowledge. This design process was also iterative, involving collaborative decision-making between the clinical and app development teams. Any discrepancies between the different sources of data from evidence-based guidelines were solved in a collaborative manner and with team consensus.

#### Patient-Reported Outcomes

Approximately 2 to 4 weeks prior to elective spine surgery, patients were invited to download the app, receive structured preoperative information, and complete baseline surveys. Perioperative information was delivered based on the timing relative to the day of surgery. Postoperative surveys were automatically available to patients after discharge, and reminders were given via automated notifications on their smartphones. All items were closed questions with predefined answers. Patients received surveys that were selected or created by the spine surgeons at Duke Spine Center. These surveys were specifically designed to capture baseline and postoperative PROs via the platform. Standardized surveys that were used included the 29-item Patient-Reported Outcomes Measurement Information System (PROMIS-29), Oswestry Disability Index, and Neck Disability Index. These are the most common outcomes collected in spine surgery, and each is well validated in multiple clinical studies in quantifying the impact of spine surgery. In addition, 4 surveys—the numerical pain assessment, the lumbar fusion approach assessment, the percent pain reduction lumbar survey, and the percent pain reduction anterior cervical discectomy and fusion (ACDF) survey—were created and used by the surgical team and are defined in Multimedia Appendix 1.

### Stage 2: Evaluation of the MMS-Spine Module Feasibility Study

#### Participants and Setting

Institutional review board approval was obtained prior to beginning the study. We performed a descriptive feasibility study in which patients were prospectively invited and enrolled to participate if they were scheduled to undergo elective spine surgery at Duke University Health System. Consent was performed electronically and obtained at the time of enrollment. A total of 47 continuous patients were included in this feasibility study. Inclusion criteria were English as the primary language, availability of a smartphone, and capacity to consent. Procedures supported by the MMS-Spine module included the most common spine surgeries, such as lumbar laminectomy and discectomy, lumbar fusion, and ACDF. Patients who did not have a phone or could not use one could assign a family member as a caregiver to operate the app on their behalf. After identification of patients via weekly operating room schedule reviews, patients were invited to download MMS via email. For this study, patients were considered engaged with and benefiting from MMS if they had downloaded and logged in to the app. Informed consent was obtained, and each patient went through a brief, standardized walk-through orientation within the app.

#### Data Collection and Analysis

Two members of the research team independently reviewed the results of the outcomes data, patient responses, and associated electronic health record data. Descriptive statistics for the surveys were calculated using Google Sheets (Google Corp) and SAS 9.4 (SAS Institute Inc). Continuous variables were reported as means, standard deviations, medians, first quartiles, third quartiles, minimum values, and maximum values. Categorical variables were reported as numbers and percentages.

Data gathered throughout the entirety of the patient’s engagement with MMS from this cohort were collected and stored securely via Amazon Web Services. Measures that were continually collected included the number of account sign-ins, task completion, the addition of a caregiver or caregivers, the device used to access MMS, and the frequently asked questions (FAQs) viewed. Additional data gathered at specific time points included PROs, collected through surveys and patient feedback regarding their experience with MMS. The PROs were requested prior to the surgery, once the patient’s procedure was added to MMS, and at various time points that were set based on when the procedure was completed. These postoperative time points included 6 weeks, 3 months, 6 months, and 12 months. Patient feedback was collected 30 days postoperatively. MMS also automatically sends push notifications to patients for tasks at various time points to gather data (eg, appointment confirmation, completion of preoperative screening, etc). This is designed to reduce the burden on the clinical staff and allow for more consistent and predictable follow-up. Descriptive statistics for the self-administered survey completion were also collected.

## Results

### Stage 1: Consultation With Experts and Intervention Design

**Figure 3:**
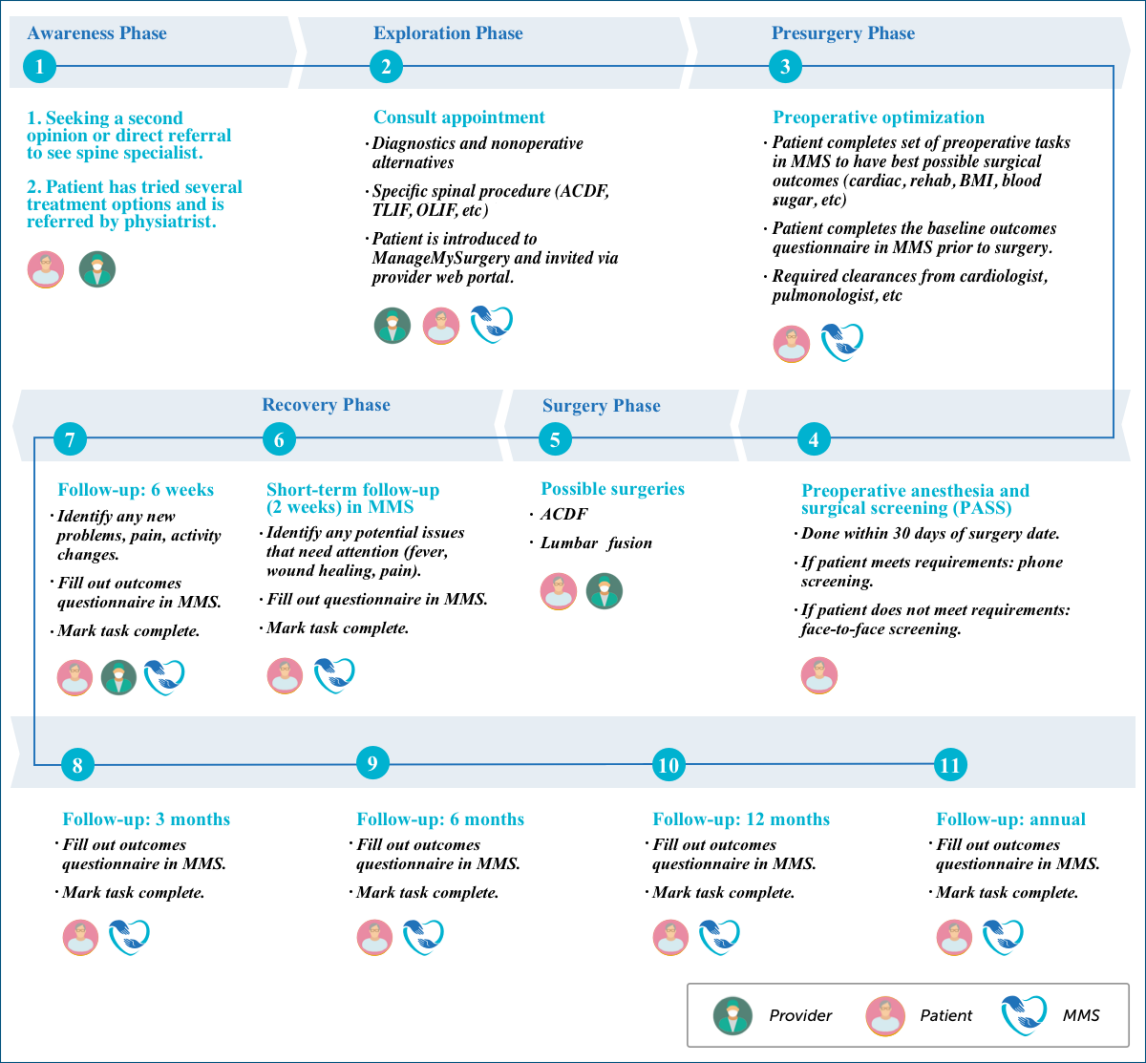
Step-by-step road map of the MMS-Spine patient care pathway. ACDF: anterior cervical discectomy and fusion; MMS: ManageMySurgery; OLIF: oblique lateral interbody fusion; rehab: rehabilitation; TLIF: transforaminal lumbar interbody fusion.

**Figure 4:**
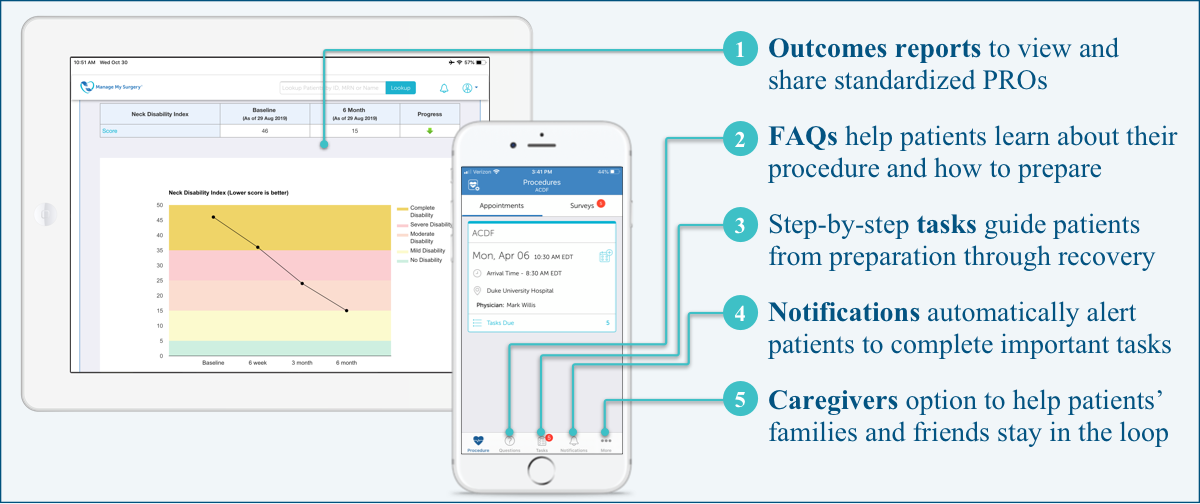
Functionality of the MMS-Spine app. FAQs: frequently asked questions; PROs: patient-reported outcomes.

Key features and design elements were used to develop a clinically seamless workflow in MMS-Spine (Figure 3). The app was designed to serve as a virtual patient navigator through the various phases of the surgical journey, from awareness to exploration, presurgery, surgery, and ultimately, recovery. Patients can self-report their outcomes and access FAQs, receive notifications, and connect to a variety of multimedia resources to educate them about their procedure and ways in which they can prepare for and recover from their surgery in order to optimize outcomes (Figure 4).

One of the key challenges was the need to adapt technical medical language when communicating within a multidisciplinary team. The scientific writing team was critical to ensuring all content conveyed complex medical knowledge at an appropriate reading comprehension level. After continuous refinement, we arrived at a viable app that guides the patient throughout the preoperative, perioperative, and postoperative periods and serves to ease the anxiety commonly encountered during surgical procedures. Additionally, by leveraging analysis of task completion and PRO results, the app can assist in identifying patients who may need attention sooner or those who do not need to be seen at all. For example, if a patient has not completed any of their preoperative tasks, they are less likely to be engaged in their upcoming procedure and potentially more likely to have a poor outcome or a complication.

### Stage 2: Evaluation of the MMS-Spine Module Feasibility Study

#### Patient Characteristics

A total of 47 patients e-consented and participated in the feasibility study. Patients from 5 spine surgeons contributed to the study. Of the 47 patients, 21 (45%) underwent lumbar fusion and 26 (55%) underwent ACDF. The median age was 59.0 (range 33-77) years, and 22 of the 47 patients (47%) were women, 26 (55%) had commercial insurance, and 40 (85%) had surgery on 1 to 3 spinal levels (Table 1). A total of 17 of the 47 patients (36%) added a caregiver (friend or family member), of which 7 (41%) logged in (Table 2). Compared with the patients who underwent lumbar fusion, patients who underwent ACDF were younger (56.4 years vs 62.3 years), more frequently female (13/26, 50% vs 9/21, 43%), used more commercial insurance (17/26, 65% vs 9/21, 43%), used fewer iOS phones (17/26, 65% vs 16/21, 76%), added more caregivers (17/26, 65% vs 13/21, 62%), and had fewer patients who did not view any FAQs (10/26, 38% vs 14/21, 67%) (Table 1 and Table 2).

**Table 1:** Patient characteristics by procedure type (N=47).

Patient characteristics		ACDF^a^ (n=26)		Lumbar fusion (n=21)		Total (N=47)	
**Age at surgery (years)**							
	Age (years), mean (SD)		56.4 (9.3)		62.3 (12.2)		59.0 (11.0)
	Age (years), median (IQR)		58.5 (50.3-62.0)		63.0 (56.0-72.0)		59.0 (52.5-67.0)
	Age (years), range		37.0-73.0		33.0-77.0		33.0-77.0
**Gender, n (%)**							
	Female		13 (50)		9 (43)		22 (47)
	Male		13 (50)		12 (57)		25 (53)
**Payor group, n (%)**							
	Commercial		17 (65)		9 (43)		26 (55)
	Medicare		6 (23)		11 (52)		17 (36)
	Other		3 (12)		1 (5)		4 (9)
**Procedure, n (%)**							
	ACDF		25 (96)		1 (5)		26 (55)
	ALIF^b^		0		1 (5)		1 (2)
	Lumbar laminectomy		0		3 (14)		3 (6)
	Posterior cervical fusion		1 (4)		1 (5)		2 (4)
	SI^c^ fusion		0		1 (5)		1 (2)
	TLIF^d^ or PLIF^e^		0		13 (62)		13 (28)
	XLIF^f^		0		1 (5)		1 (2)
**Surgery levels, n (%)**							
	1		6 (23)		8 (38)		14 (30)
	2		10 (38)		6 (29)		16 (34)
	3		6 (23)		4 (19)		10 (21)
	4		2 (8)		1 (5)		3 (6)
	5		0		1 (5)		1 (2)
	8		1 (4)		0		1 (2)
	Missing		1 (4)		1 (5)		2 (4)

^a^ACDF: anterior cervical discectomy and fusion.

^b^ALIF: anterior lumbar interbody fusion.

^c^SI: sacroiliac.

^d^TLIF: transforaminal lumbar interbody fusion.

^e^PLIF: posterior lumbar interbody fusion.

^f^XLIF: extreme lateral interbody fusion.

**Table 2:** Patient usage results by procedure type (N=47).

Usage		ACDF^a^ (n=26)	Lumbar fusion (n=21)	Total (N=47)
**Patient sign-in count, n (%)**				
	1-4	23 (88)	16 (76)	39 (83)
	5-9	2 (8)	3 (14)	5 (11)
	10+	1 (4)	2 (10)	3 (6)
Patient sign-in count, mean (SD)		3.0 (4.7)	3.5 (2.8)	3.2 (3.9)
Patient sign-in count, median (IQR)		2 (1-3)	2 (2-4)	2 (1-4)
**Caregiver added, n (%)**				
	No	17 (65)	13 (62)	30 (64)
	Yes	9 (35)	8 (38)	17 (36)
Added caregivers that logged in, n (%)		3 (33)	4 (50)	7 (41)
**Device, n (%)**				
	iOS	17 (65)	16 (76)	33 (70)
	Android	9 (35)	2 (10)	11 (23)
	Web or notifications off	0	3 (14)	3 (6)
**Viewed questions, n (%)**				
	0	10 (38)	14 (67)	24 (51)
	1-10	3 (12)	2 (10)	5 (11)
	11-20	5 (19)	0	5 (11)
	21-30	4 (15)	4 (19)	8 (17)
	31-40	1 (4)	1 (5)	2 (4)
	51-60	2 (8)	0	2 (4)
	81-90	1 (4)	0	1 (2)
Viewed questions, mean (SD)		16.0 (20.8)	7.4 (12.5)	12.2 (17.9)
Viewed questions, median (IQR)		12.5 (0.0-22.5)	0.0 (0.0-9.0)	0.0 (0.0-21.5)
Viewed questions, range		0.0-85.0	0.0-36.0	0.0-85.0

^a^ACDF: anterior cervical discectomy and fusion.

#### App Use

**Figure 5:**
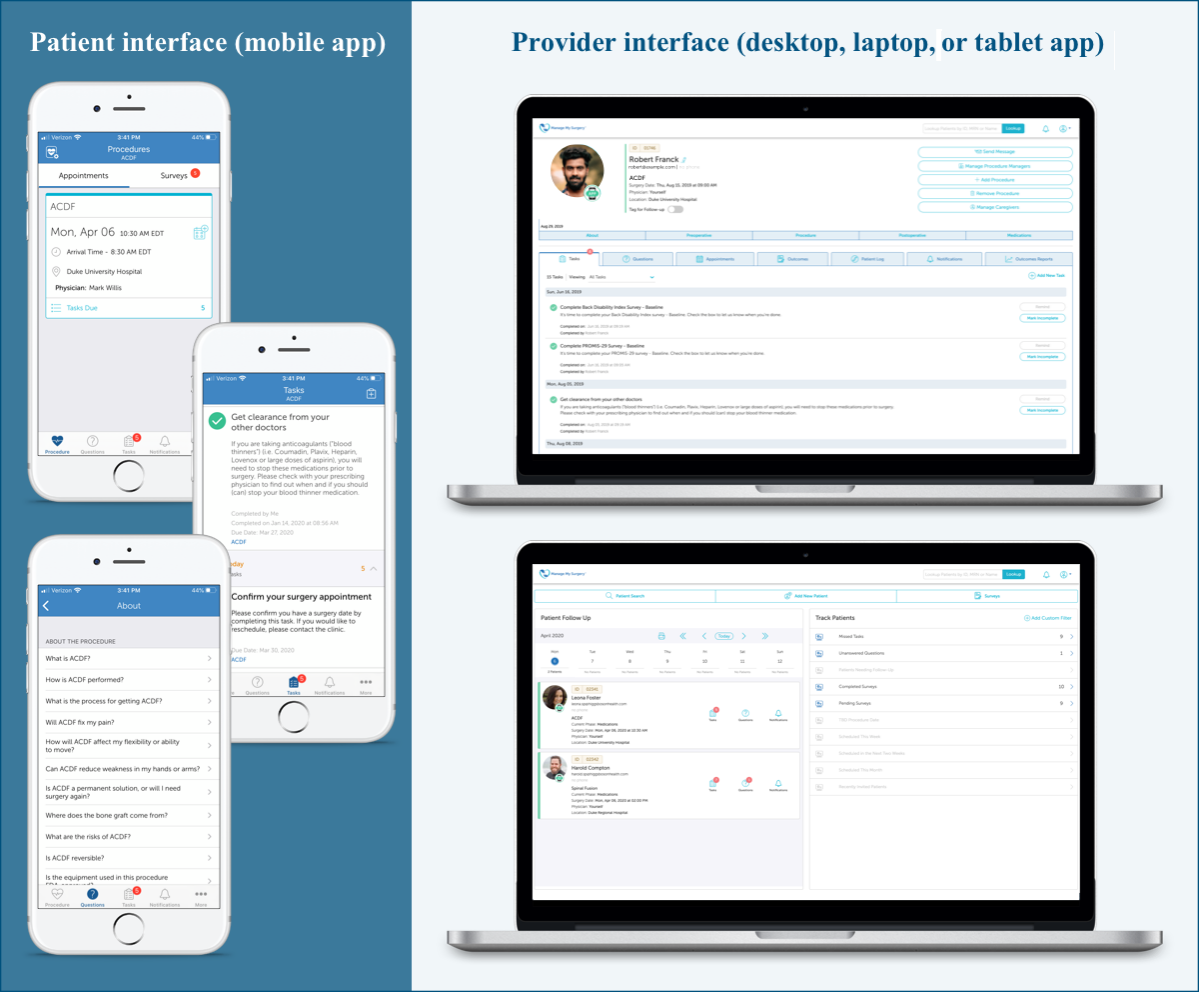
Screenshot of the MMS-Spine patient (mobile app) and provider (desktop, laptop, or tablet) interfaces.

During the feasibility study, 100% (47/47) of patients interacted with the app by downloading and logging in, meeting our definition of feasibility (Table 2). Screenshots of the patient- and provider-facing interface of the MMS-Spine app are shown in Figure 5. A total of 33 of the 47 patients (70%) used an iOS phone or tablet device to access the app, 11 (23%) used an Android device, and 3 (6%) used a web browser or phone with notifications turned off (Table 2).

The median number of log-ins into the app was 2, with 83% (39/47) of patients signing in 1 to 4 times (a log-in was defined as any time the patient input their username and password to access their account) (Table 2). Among the 47 patients, the top 3 most-viewed FAQs were (1) How soon can I start driving again after the procedure? (20/47, 43%); (2) What serious symptoms should I watch for during my recovery? (16/47, 34%); and (3) How will I feel after the surgery? (16/47, 34%) (Table 3).

Of the 47 patients, 24 (51%) provided feedback on the MMS-Spine app. Among these 24 patients, 12 (50%) found the app very helpful and 9 (38%) found the app somewhat helpful in preparing for their surgery. In addition, 8 of the 24 respondents (33%) found it very helpful and 8 (33%) found it somewhat helpful in recovering from their surgery. A total of 23 of the 24 respondents (96%) stated that they would recommend MMS to a friend or family member (Table 4).

**Table 3:** Most viewed frequently asked questions by procedure type (N=47).

Procedure type and question		Views, n (%)
**ACDF^a^ (n=26)**		
	What are the risks of ACDF?	13 (50)
	What is ACDF?	8 (31)
**Lumbar fusion (n=21)**		
	How will a spinal fusion affect my flexibility or ability to move?	5 (24)
	What are the risks of spinal fusion?	4 (19)
	What is the process for getting a spinal fusion?	4 (19)
**Both ACDF and lumbar fusion (N=47)**		
	How soon can I start driving again after the procedure?	20 (43)
	What serious symptoms should I watch for during my recovery?	16 (34)
	How will I feel after the surgery?	16 (34)
	How long will I be in the hospital?	13 (28)
	Are there restrictions on eating or drinking after the procedure?	13 (28)
	How long will I be in the hospital?	13 (28)

^a^ACDF: anterior cervical discectomy and fusion.

**Table 4:** MMS patient feedback survey results (N=47).

Feedback		ACDF^a^ (n=26)	Lumbar fusion (n=21)	Total (n=47)
Survey completion, n (%)^b^		14 (54)	10 (48)	24 (51)
**MMS^c^ helpfulness in procedure preparation, n (%)^d,e^**				
	1	6 (43)	6 (60)	12 (50)
	2	7 (50)	2 (20)	9 (38)
	3	1 (7)	2 (20)	3 (13)
	4	0 (0)	0 (0)	0 (0)
	5	0 (0)	0 (0)	0 (0)
**MMS helpfulness in procedure recovery, n (%)^d,e^**				
	1	5 (36)	3 (30)	8 (33)
	2	5 (36)	3 (30)	8 (33)
	3	4 (29)	3 (30)	7 (29)
	4	0 (0)	1 (10)	1 (4)
	5	0 (0)	0 (0)	0 (0)
**MMS recommendation to a family or friend, n (%)^d,e^**				
	Yes	13 (93)	10 (100)	23 (96)
	No	1 (7)	0 (0)	1 (4)

^a^ACDF: anterior cervical discectomy and fusion.

^b^Signifies the number of surveys completed out of the entire cohort of 47 patients.

^c^MMS: ManageMySurgery.

^d^Signifies the number of specified responses out of the total number of 24 surveys completed.

^e^Key: 1=very helpful, 2=somewhat helpful, 3=neither helpful nor unhelpful, 4=somewhat unhelpful, 5=very unhelpful.

**Figure 6:**
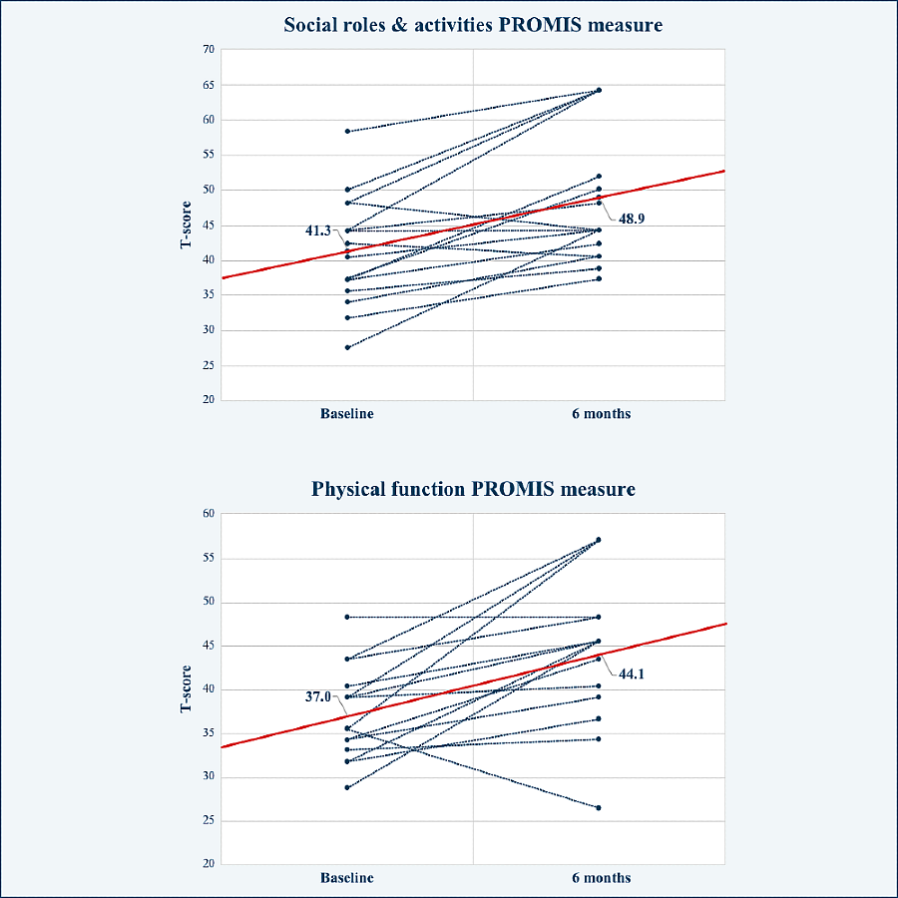
PROMIS-29 outcome measures for social roles and activities and for physical function compared at baseline and 6-month time points for 16 members of the cohort. PROMIS: Patient-Reported Outcomes Measurement Information System.

A total of 38 of 47 patients (81%) completed their baseline preoperative PROMIS-29 outcomes. At 6 weeks, 3 months, 6 months, and 12 months postoperatively, the number of patients who completed PROMIS-29 surveys out of the 47 total patients was 13 (28%), 8 (17%), 6 (13%), and 1 (2%), respectively. A total of 14 of 47 patients (30%) completed at least 1 PROMIS-29 survey during the postoperative period, with the highest response rate at 6 weeks (13/47, 28%) (Table 5). The MMS-Spine app has the capability of converting PROMIS-29 T-score data into graphs (Figure 6) or visualizations to clearly compare a patient’s baseline and postoperative outcome measures at specified time points. Figure 6 gives one example of this by comparing T-scores at baseline and 6 months post procedure for 16 cohort members in the PROMIS-29 domain of social roles and activities and the domain of physical function, showing an average score increase from 41.3 (mild) to 48.9 (normal) and 37.0 (moderate) to 44.1 (normal), respectively. For a full list of T-scores collected from baseline through 12 months post operation across all PROMIS domains, see Multimedia Appendix 2.

**Table 5:** Completion rate of self-administered Back Disability Index, Neck Disability Index, percent pain reduction, and PROMIS-29 surveys.

Procedure and survey		Baseline	Postoperative time point				
			6 weeks	3 months	6 months	12 months	At least one completed
**ACDF^a^, n (%) (n=26)**							
	Neck Disability Index	22 (85)	5 (19)	3 (12)	3 (12)	0 (0)	6 (23)
	Percent pain reduction	N/A^b^	5 (19)	4 (15)	3 (12)	0 (0)	7 (27)
**Lumbar fusion, n (%) ** **(n=21)**							
	Back Disability Index	15 (71)	6 (29)	3 (14)	3 (14)	1 (5)	6 (29)
	Numerical pain assessment survey	16 (76)	6 (29)	3 (14)	3 (14)	1 (5)	7 (33)
	Lumbar fusion approach assessment	N/A	7 (33)	2 (10)	3 (14)	1 (5)	7 (33)
	Percent pain reduction	N/A	5 (24)	4 (19)	5 (24)	1 (5)	6 (29)
**Both ACDF and lumbar fusion, n (%) ** **(N=47)**							
	PROMIS-29^c^	38 (81)	13 (28)	8 (17)	6 (13)	1 (2)	14 (30)

^a^ACDF: anterior cervical discectomy and fusion.

^b^N/A: not applicable.

^c^PROMIS-29: 29-item Patient-Reported Outcomes Measurement Information System.

## Discussion

### Principal Findings and Future Work

Our results demonstrate that it is feasible to use a novel mobile health app (MMS-Spine) to engage patients in their spine surgery journey. The majority of patients tracked outcomes, completed tasks, and engaged with the FAQs at some point through their surgical journey, decreasing the burden on clinical and research staff.

This study is one of the first to report a patient-centered approach to developing a smartphone-based platform for patients undergoing elective spine surgery. Previous health care applications have primarily focused on chronic medical conditions [2] or symptom monitoring [8], and there has been little research regarding how the applications can be integrated into clinical practice. Prior mobile app studies have shown effectiveness in promoting behaviors for surgical recovery by recording patient adherence to postsurgical instructions, providing rehabilitation exercises, and monitoring medication use [4,9,10]. MMS incorporates a focus on short-term behavior change, as demonstrated in this feasibility study for spine surgery.

Engagement, defined as how a user interacts with technology and their emotional response to it, was a key metric of success. There are a number of metrics to evaluate engagement, from user satisfaction to more complex user engagement scores [11] and, on the commercial side, net promoter scores [12]. Because of the relatively short duration of use for this app, we decided to focus more on short-term user experience metrics and tended to avoid longer-term or patient loyalty metrics such as net promoter scores. Thus, for this initial feasibility study, we tracked overall patient satisfaction with the app and patient completion of key PROs. Overall, 96% of patients (23/24) found the app easy to use and would recommend it to a friend or family member. Additionally, participants expressed that the clear, concise presentation of information and the timely tasks and notifications were beneficial. Finally, we noted that patient engagement was extremely high prior to surgery, with 38 of 47 patients (81%) completing their baseline preoperative PROMIS-29 outcomes. During the postoperative period, however, only 14 of the 47 patients (30%) completed at least one postoperative PROMIS-29 survey, indicating that additional strategies are needed to maintain patient engagement after the surgical event.

Future iterations will incorporate strategies to improve patient engagement and the number of postoperative outcomes that are collected. Several strategies for increasing patient engagement are possible when designing mobile health apps, including design-thinking techniques, improved notifications and messaging, and the incorporation of opportunities for feedback [13]. We aim to increase engagement by both increasing log-in rates and by improving our reminder and notification system. To increase the initial log-in rate, patients who were invited but did not log in to the program will be polled, and their reasons for not using MMS will be analyzed and addressed. In order to improve reminders and notifications, patients who began using MMS but did not complete the long-term follow-up surveys will be polled to better understand their reasons for not returning to the app. App updates will be designed and implemented with this feedback in mind. Preliminary options to increase follow-up response include sending reminders via additional mediums, collecting PROs via email and text from patients who have not downloaded the app, using artificial intelligence to optimize notification delivery, and sending messages to patients who have been less engaged around the time of their procedure. Finally, we plan to provide greater value to patients. To accomplish this, we agree with the conclusions of Bombard and colleagues [14] that making patients feel heard is hugely important to maintaining their engagement with the platform. We plan to integrate messaging or system alerts for patients with suboptimal outcomes who may need more attention from a care provider. The downside to this approach is the higher technical cost and potential to overwhelm providers. We are also considering sharing outcome reports directly with patients at certain milestones. This can naturally incentivize future survey completion, as patients have direct knowledge about their health. Sharing data and comparisons with national averages with patients would be at the discretion of the provider and require contextualized explanations of PROs to maximize value and understanding by the patient.

Enhancing usability and engagement is another crucial element of the effectiveness of mobile apps in health care [13]. Higher-stress interventions, such as surgery, may lead to higher user engagement (measured through log-ins and repeated use), which has been associated with better health outcomes [10,15]. However, patients will not actually use a beneficial tool with a poor user experience. Consumer expectations for mobile health care apps are high and only increasing; a recent survey demonstrated that peoples’ tolerance for poorly performing apps has reduced over time, even just in recent years [16,17]. Thus, we have been careful to maintain a collaborative workflow between clinicians, developers, and scientific writers, with a constant focus on functional design. Development of future features that continue improving usability and engagement will iterate on these foundational principles and simultaneously add value to patients by enabling them to stay on care pathways that lead to the best possible surgical outcomes.

Usability from the provider perspective is also crucial in health care app development. Recent studies have highlighted the importance of user experiences for both the patient and provider [18]. As such, we optimized the app for ease of use for the provider while also providing maximum flexibility to adapt to new procedures and surgeries. The MMS app is built as a platform that can be used for any interventional or surgical procedure, with the focus of the current feasibility study being spine surgery. It can accommodate many different patient care pathways, is highly configurable to fit a health system’s workflow, and facilitates the transition of therapy and care. MMS was made available to patients through a web application, but for increased usability and adoption rate, both iOS and Android phone apps are also available and were primarily used in this feasibility study. Finally, MMS was designed to be compatible with any electronic health record, thus facilitating implementation for the hospital. Moreover, MMS is available with out-of-the-box content that is fully customizable to meet the client’s needs.

Despite the rapid expansion of the field of mobile health, there have been few studies in surgical patients, especially spine surgery. Any studies focusing on ambulatory surgeries have focused entirely on postoperative care. For example, mobile apps have been demonstrated to reduce 30-day readmission rates in ambulatory breast reconstruction [19] and reduce in-person follow-up for lumbar discectomies [20]. Taken together, these data suggest that a comprehensive app that includes preoperative, perioperative, and postoperative values could be effective on a larger scale. MMS-Spine is unique in the breadth and variety of information it provides to the patient and caregiver. The proper use of prespecified tasks and notifications allows one to rapidly identify which patients are off track and anticipate problems that might require patient-provider communication. In an increasingly telemedicine- and efficiency-focused US health care system, patients often come inadequately prepared for their surgical procedure or leave the surgical center experiencing symptoms that were previously attended to by the health care team. Without information and risk awareness, patients may delay seeking care, experience unnecessary anxiety, or seek unnecessary care, which can all lead to increased costs. These scenarios put additional strain on the health care system through the need for potentially expensive unplanned hospital readmissions, corrective procedures for complications that could have been addressed more easily at an earlier stage, or the burdening of medical personnel with hospital visits for minor complaints that could have been addressed remotely. Research regarding digital health solutions remains scarce, and more studies with larger sample sizes and longer follow-ups are needed to evaluate the impact of mobile health apps on surgical outcomes.

### Limitations

Participation bias may have influenced the feasibility study. For example, all study participants were computer literate and had ready access to smartphones. We considered this limitation by alternatively developing the application as a web application that could run on a desktop or laptop (using modern browsers, including Chrome, Firefox, Safari, and Internet Explorer). In addition, the ability to add a caregiver helps minimize the barrier to technology adoption, as usually one or more members of a family have access to a smartphone (>80% of the US population [21]). The data collection was limited to counting the number of log-ins and not the number of times the app was opened. This number could be much greater than the number of log-ins indicates because 1 log-in allows a patient to access their account for up to 2 weeks when the smartphone has an enabled locking mechanism. Additionally, our data are limited to descriptive usage statistics, and future comparative studies will need to be performed to examine the effects of MMS usage on clinical outcomes and health care resource utilization. Finally, further refinements of the app may be needed to help engage patients who are less familiar with technology.

### Conclusions

In summary, we used a patient-centered approach to develop one of the first comprehensive smartphone apps for patients undergoing elective spine surgery. This study summarizes the sequential and iterative process of developing the MMS-Spine app, which is aimed at navigating the spine surgery journey. Feasibility testing provided useful information regarding users’ experiences with the app. The optimized version of the app will be ready for formal testing in a larger randomized clinical study to establish its cost-effectiveness and effect on patients’ self-management skills and long-term outcomes.

## Appendix

Multimedia Appendix 1 Additional survey instruments created and used by Duke Spine Center to assess patient pain during recovery.

Multimedia Appendix 2 PROMIS-29 outcomes results (self-administered and proxied) at individual and aggregated timepoints.

## Abbreviations

ACDF: anterior cervical discectomy and fusion
BCT: behavior change technique
FAQ: frequently asked question
MMS: ManageMySurgery
MMS-Spine: ManageMySurgery spine surgery module
PRO: patient-reported outcome
PROMIS-29: 29-item Patient-Reported Outcomes Measurement Information System
