# Supplementary material for: A Smartphone App With a Digital Care Pathway for Patients Undergoing Spine Surgery: Development and Feasibility Study
Source: JMIR Perioper Med. 2020 Oct 16;3(2):e21138. doi: 10.2196/21138 (PMC7709850; doi:10.2196/21138)
Supplement: Multimedia Appendix 1 [file periop_v3i2e21138_app1.docx]

**Multimedia Appendix 1.** Additional survey instruments created and used by Duke Spine Center to assess patient pain during recovery.

| **Survey** | **Question** | **Answer Choices** |
| --- | --- | --- |
| **Numerical Pain Assessment** | Over the last 24 hours, please rate your pain in your LEFT LEG | N/A, 0 being no pain, 1, 2, 3, 4, 5, 6, 7, 8, 9, 10 being worst pain imaginable |
|  | Over the last 24 hours, please rate your pain in your RIGHT LEG | N/A, 0 being no pain, 1, 2, 3, 4, 5, 6, 7, 8, 9, 10 being worst pain imaginable |
|  | Over the last 24 hours, please rate your pain in your LOWER BACK | N/A, 0 being no pain, 1, 2, 3, 4, 5, 6, 7, 8, 9, 10 being worst pain imaginable |
|  | Over the last 24 hours, please rate your OVERALL pain | N/A, 0 being no pain, 1, 2, 3, 4, 5, 6, 7, 8, 9, 10 being worst pain imaginable |
| **Lumbar Fusion Approach Assessment** | Since your surgery, have you experienced any new front thigh/groin sensory changes (numbness, tingling)? If so, how much would you estimate? | N/A, 0 (no thigh/groin numbness/tingling), 1, 2, 3, 4, 5, 6, 7, 8, 9, 10 (severe thigh/groin numbness/tingling) |
|  | Since your surgery, have you experienced any lower leg sensory changes (numbness, tingling)? If so, how much would you estimate? | N/A, 0 (no lower leg numbness/tingling) - 10 (severe lower leg numbness/tingling), in increments of 1. |
|  | Since your surgery, have you experienced any new front thigh/groin pain? If so, how much would you estimate? | N/A, 0-10; 0 being no thigh/groin pain and 10 being severe thigh/groin pain. |
|  | Since your surgery, have you experienced any new weakness in bending your leg (hip, knee or foot)? If so, how much would you estimate? | N/A, 0 (no weakness) -10 (complete and continued weakness), in increments of 1 |
|  | Considering your complete experience with your surgery, how likely would you be to recommend the same surgery to a friend or colleague? | 0 (very unlikely) -10 (very likely), in increments of 1. |
| **Percent Pain Reduction Lumbar Survey** | Compared to before the surgery, how much has your LEFT LEG pain improved? | N/A, 0 (no improvement) -100% (complete improvement), in increments of 10% |
|  | Compared to before the surgery, how much has your RIGHT LEG pain improved? | N/A, 0 (no improvement) -100% (complete improvement), in increments of 10% |
|  | Compared to before the surgery, how much has your LOWER BACK pain improved? | N/A, 0 (no improvement) -100% (complete improvement), in increments of 10% |
|  | Compared to before the surgery, how much has your OVERALL pain improved? | N/A, 0 (no improvement) -100% (complete improvement), in increments of 10% |
|  | Apart from your regular day-to-day medications, were you able to stop or reduce the amount of “as needed” pain medication that you only use if the pain is really bad?” | 1) N/A, 2) Yes, stopped completely. 3)Yes, reduced by more than 50% 4) Yes, reduced by less than 50%. 5) No, no change at all. |
|  | If taking pain medications prior to surgery, what percent have you been able to decrease them since surgery? | N/A, 0 (no reduction in pain medication) -100% (completely weaned off pain medications), in increments of 10% |
| **Percent Pain Reduction ACDF Survey** | Compared to before the surgery, how much has your LEFT ARM pain improved? | N/A, 0 (no improvement) -100% (complete improvement), in increments of 10% |
|  | Compared to before the surgery, how much has your RIGHT ARM pain improved? | N/A, 0 (no improvement) -100% (complete improvement), in increments of 10% |
|  | Compared to before the surgery, how much has your NECK pain improved? | N/A, 0 (no improvement) -100% (complete improvement), in increments of 10% |
|  | Compared to before the surgery, how much has your OVERALL pain improved? | N/A, 0 (no improvement) -100% (complete improvement), in increments of 10% |
|  | Apart from your regular day-to-day medications, were you able to stop or reduce the amount of “as needed” pain medication that you only use if the pain is really bad? | 1) N/A 2) Yes, stopped completely. 3)Yes, reduced by more than 50% 4) Yes, reduced by less than 50%. 5) No, no change at all. |
|  | If taking pain medications prior to surgery, what percent have you been able to decrease them since surgery? | N/A, 0 (no improvement) -100% (complete improvement), in increments of 10% |
