# Supplementary material for: A Smartphone App With a Digital Care Pathway for Patients Undergoing Spine Surgery: Development and Feasibility Study
Source: JMIR Perioper Med. 2020 Oct 16;3(2):e21138. doi: 10.2196/21138 (PMC7709850; doi:10.2196/21138)
Supplement: Multimedia Appendix 2 [file periop_v3i2e21138_app2.docx]

**Multimedia Appendix 2.** PROMIS-29 outcomes results (self-administered and proxied) at individual and aggregated timepoints.

| Health Measure | Baseline:  T-score (SD) | 6 Week:  T-score (SD) | 3 Month:  T-score (SD) | 6 Month:  T-score (SD) | 12 Month:  T-score (SD) | All Post-Op:  T-score (SD) |
| --- | --- | --- | --- | --- | --- | --- |
| Anxiety | 54.9 (3.2) | 48.1 (4.6) | 49.0 (4.3) | 51.7 (3.9) | 47.0 (4.5) | 50.1 (4.2) |
| Depression | 49.9 (4.2) | 49.1 (4.3) | 50.0 (4.3) | 48.8 (4.5) | 45.0 (4.7) | 48.7 (4.5) |
| Fatigue | 56.9 (2.7) | 56.4 (2.6) | 55.0 (2.6) | 55.4 (2.8) | 55.9 (2.4) | 55.1 (2.7) |
| Pain Interference | 66.6 (2.2) | 61.3 (2.6) | 58.2 (2.5) | 60.1 (2.3) | 59.2 (1.9) | 59.8 (2.5) |
| Sleep Disturbance | 58.0 (3.4) | 57.1 (3.5) | 55.5 (3.5) | 55.7 (3.6) | 60.8 (3.3) | 54.0 (3.5) |
| Physical Function | 36.2 (2.5) | 38.1 (2.9) | 41.6 (3.2) | 43.8 (3.3) | 43.5 (2.6) | 42.2 (3.3) |
| Social Roles & Activities | 40.9 (2.4) | 42.5 (2.4) | 46.9 (2.9) | 48.5 (2.9) | 48.1 (2.3) | 47.4 (2.9) |
| Patient Count: | 38 | 13 | 13 | 18 | 2 | 31 |

^a^ PROMIS-29 measures are on the T-score metric with a mean of 50 and standard deviation of 10 in the general population.
